# Climate Change and Infectious Diseases: Evidence from Highly Vulnerable Countries

**Published:** 2019-12

**Authors:** Asim ANWAR, Sajid ANWAR, Muhammad AYUB, Faisal NAWAZ, Shabir HYDER, Noman KHAN, Imran MALIK

**Affiliations:** 1. Department of Management Sciences, COMSATS University Islamabad, Attock Campus, Punjab, Pakistan; 2. School of Business, University of Sunshine Coast, Maroochydore DC, Australia

**Keywords:** Climate change, Human infectious diseases, Temperature, Population density

## Abstract

**Background:**

Climate change is an alarming challenge for humanity at large due to its mediating role in emergence and spread of infectious diseases like cholera and malaria. This study was conducted to examine the effect of climate change and some socio-economic factors on incidence of infectious diseases.

**Methods:**

We used country level panel data over the 1990–2017 period using panel ARDL-PMG technique on highly affected countries from climate change.

**Results:**

There is a long run co-integrating relationship among climate change, socio-economic factors and prevalence of infectious diseases. Climate change, as measured by the temperature, is contributing to the spread of infectious diseases.

**Conclusion:**

This is the first study giving evidence of the impact of climate change on incidence of infectious diseases as can be seen from highly vulnerable countries to climate change. It is recommended to improve the level of education along with public health and town planning to reduce the incidence of infectious diseases.

## Introduction

The human economic activity has raised not only standard of living in most countries but also has raised global concerns in terms of climate change (1). Climate change refers to continuous shift in weather patterns over a long period with respect to growing human activities, biotic processes and plate tectonics. The Intergovernmental Panel on Climate Change (IPCC) has delineated an increase of 0.74°C in worldwide average surface temperature in 20^th^ century and it continues to move up at an average of 1.5–5.8 °C in the 21^st^ century (2, 3). The global warming and consequent change in climate is holding multifaceted challenges to the world with reference to perilous conditions of human health perpetrated by the infectious diseases (4).

Many factors like socio-economic conditions, availability of health care facilities, and inner human immunity along with weather conditions determine the spread of infectious diseases (5–7) (5). Numerous infections carrier like vector organisms, reservoir species of non-human agents and pathogens have particular sensitivity to weather patterns (8). Climate change provides vulnerable conditions for diverse infectious diseases borne by water, air and food (9). The process of carrying these diseases is done by three active agents i.e. pathogen, host and a vulnerable transmission environment (10, 11). Climate change either directly affects the life cycle, survival and reproduction of pathogens or indirectly influences these activities by manipulating the local conditions like environments and other competitors for the pathogens (12). Moreover, temperature plays a mediating role for survival and development of pathogens like maximum temperature ranging from 22–23 °C is highly vulnerable for mosquitoes’ development and survival while minimum temperature ranging from 25–26 °Cis required for spread of Japanese Encephalitis virus (JEV) and also plays a key role in ecology of JEV (13, 14). However, excessive heat also raises the mortality rates for some pathogens (15, 16). Similarly, a rise in temperature can also influence the reproduction and extrinsic incubation period (EIP) of pathogens like the EIP for P. falciparum reduces from 26 days at 20 °C to 13 days at 25 °C (17, 18).

The research highlighted an ever increasing role of warm and unstable climate change in accelerating the worldwide emerging, resurging and redistribution of infectious diseases (13, 19). The infectious diseases transmitted through insects like dengue, cholera, malaria are extremely responsive to climate change followed by diseases transmitted through water, food and soil (13, 16). For survival, the V. Cholerae need an optimum temperature and physicochemical circumstances (salinity, pH, humidity etc.) (20). Nevertheless, a manifestation has been also seen to resist the suboptimal conditions via specific collaboration of the bacterium with aquatic plants or animals like oysters, copepods and crabs that helps the pathogen to persist for longer time spans in aquatic habitats (21, 22). Moreover, weather conditions like an increasing temperature in environmental or sea surface conditions help plankton bloom (20). Likewise crowded living conditions and poor access to water and sanitation services along with environmental degradation in developing countries make the environment conducive to cholera incidence (23, 24). While in case of measles both extreme weathers i.e. hot and cold lowers its probability of incidence, however, any change resulting in the moderation of weather caused by climate change may increase its incidence (25, 26). Furthermore, measles is a contagious disease, therefore more likely to spread as population density increases (27).

While a number of studies have considered the nexus amid infectious diseases and climate change, only a relatively small number of studies have utilized the data that covers a sufficiently long time-span. The time-span is particularly important for countries that are exposed to the highest climate change risk. This paper provides empirical evidence that promotes the understanding of the ecological and socioeconomic drivers of cholera and measles’ outbreak in some developing countries that are severely affected by the climate change reported by German watch Organization. The analysis presented in this paper could also contribute to a more accurate prediction of the spread of epidemic diseases thereby allowing the relevant authorities to take more effective steps in infectious disease control.

## Methods

### Description of Variables and Empirical Model

Our empirical model is based on a study (28), extended by others (29, 30). The latter studies examined the nexus between infectious diseases and climate change. We extended framework of (29, 30) by including climatic change and socioeconomic factors that can have a significant effect on infectious diseases’ incidence. The study focused on countries that are significantly affected by climate change in recent years i.e. India, Thailand, Philippines, Bangladesh, Zimbabwe and Myanmar. The empirical model is as follows:

[1] M=g(CV,D)

Where *M* denotes the persons who got treatment of infectious diseases, while, *CV* denotes the climate variable proxy by temperature. Further, *D* denotes socio-economic variables like education, income and population density.

The researchers have included socio-economic features in equation [1] aiming to have valid results. A static model was estimated where the number of patients infected in the current period is independent with reference to previous years’ patients. Most of the infectious diseases quickly spread in different time periods consisting of days, of weeks and sometimes of months. The researchers hypothesized that spreading of disease reaches to its steady-state in a period of year and therefore this model is appropriate. As available data is annual, therefore, the researchers have employed static model stated as:

[2] $$M_{\mathrm{it}}=\beta_{0}+\beta_{1}\mathrm{tem}p_{\mathrm{it}}+\delta D_{\mathrm{it}}+\varepsilon_{\mathrm{it}}$$

We used country-level panel dataset and each variable included in equation [2] refers to county *I* at time *t*. *D*_*it*_ represents socio-economic factors (income, education and population density), *temp*_*it*_ is a measure of climate change which is our main explanatory variable, and *∊*_*it*_ is the error term; which captures the effect of all omitted variables.

The researchers measured the number of patients considered per 100,000 inhabitants as a proxy for the dependent variable. Infectious diseases discussed in this study are Cholera and Measles. The Gross Domestic Product (GDP) per capita is used as a proxy for national income, population density while secondary school enrollment (has been used as a proxy for national educational level) as independent variables in the model. We used the pooled mean grouped (PMG) estimator (31) to model the panel co-integration equation. The PMG estimator assumes the long-run coefficient to identical but allows error variances and short-run coefficients to vary across groups. It is modified version of mean group (MG) estimators (32). The MG estimator uses average values of the coefficients for each group, so the MG estimator assumes the slope coefficients and error variances to be homogeneous, in this way MG is close to pooled estimators’ class(31). Following Pesaran et al (31), the long-run equation can be written as:

[3] $$M_{\mathrm{it}}=\beta_{0}+\beta_{1}\mathrm{tem}p_{\mathrm{it}}+\beta_{2}\mathrm{GD}P_{\mathrm{it}}+\beta_{3}\mathrm{Po}p_{\mathrm{it}}+\beta_{4}\mathrm{le}d_{\mathrm{it}}+\mu_{\mathrm{it}}+\varepsilon_{\mathrm{it}}$$

We used maximum two lags and conducted the ARDL (2,2,2,2) equation as follows:

[4] $$\begin{array}{l} M_{\mathrm{it}}=\Psi_{10i}\mathrm{tem}p_{\mathrm{it}}+\Psi_{11i}\mathrm{tem}p_{1,t-2}+\\ \Psi_{20i}\mathrm{GD}P_{\mathrm{it}}+\Psi_{21i}\mathrm{GD}P_{i,t-2}+\Psi_{30i}\mathrm{Po}p_{\mathrm{it}}+\\ \Psi_{31i}\mathrm{Po}p_{i,t-2}+\Psi_{40i}\mathrm{le}d_{\mathrm{it}}+\Psi_{41i}\mathrm{le}d_{i,t-2}+\\ \zeta_{i}\mathrm{tem}p_{i,t-1}+\mu_{\mathrm{it}}+\varepsilon_{\mathrm{it}} \end{array}$$

Equation for error correction form is written as follows:

[5] $$\begin{array}{l} M_{\mathrm{it}}=i_{t}(M_{i,t-1}-\zeta_{0i}-\zeta_{1i}\mathrm{tem}p_{\mathrm{it}}-\\ \zeta_{2i}\mathrm{GD}P_{\mathrm{it}}-\zeta_{3i}\mathrm{Po}p_{\mathrm{it}}-\zeta_{4i}\mathrm{le}d_{\mathrm{it}})+\\ \Psi_{11i}\mathrm{tem}p_{\mathrm{it}}+\Psi_{21i}\mathrm{GD}P_{\mathrm{it}}+\Psi_{31i}\mathrm{Po}p_{\mathrm{it}}+\\ \Psi_{41i}\mathrm{le}d_{\mathrm{it}}+\phi_{\mathrm{it}} \end{array}$$

Where

$$\begin{array}{l} i_{t}=-(1-\zeta_{i}),\;\zeta_{0i}=\mu_{i}/\varsigma_{i},\\ \;\zeta_{1i}=\Psi_{10i}+\\ \Psi_{11i}/1\varsigma_{i},\;\zeta_{2i}=\Psi_{20i}+\Psi_{21i}/1-\varsigma_{i}\\ \;\zeta_{3i}=\Psi_{30i}+\Psi_{31i}/1-\varsigma_{i},\\ \;\zeta_{4i}=\Psi_{40i}+\Psi_{41i}/1-\varsigma_{i} \end{array},$$

In equation [5], φ represents error correction term; *ζ* represents long run coefficients while Ψ show short run coefficients.

## Results and Discussion

To examine the issue of non-stationarity, we tested panel unit roots in the selected variables. Specifically, we relied on the Levin-Lin-Chu, Im-Pesaran-Shin, and Fisher-type tests (assuming homogeneity of the dynamic panel auto regression in all panels) (Table 1).Given the mixed order of integration, we first applied co-integration using Kao procedure. The results are stationary at level I(0) for temperature (Temp), education (Ed) and population density (Pop), however, Income(GDP) result is stationary at first difference I(1) on individual panel unit root testing. The statistical significance at 1%, 5%, 10% is denoted by ***, **, * respectively.

**Table 1:** Results of Panel Unit Root test

***Order of integration of Variable***	***Levin-Lin-Chu Test***		***Im-Pesaran-Shin Test***		***Fisher-type Test***	
	**t-*Statistics***	**P-*value***	***Z-t-tilde-bar***	**P-*value***	***Pm***	**P-*value***
Patients I(0)	−2.8276^***^	0.0023	−3.1462^***^	0.0008	7.3094^***^	0.0000
Pop I(0)	−5.2001^***^	0.0000	−3.4791^***^	0.0003	11.1741^***^	0.0000
Temp I(0)	−5.2280^***^	0.0000	−5.8502^***^	0.0000	3.9733^***^	0.0000
GDP I(1)	−2.6553^***^	0.0043	−3.1202^***^	0.0009	1.8430^**^	0.0327
Ed I(0)	−2.7019^***^	0.0034	−2.3297^***^	0.0099	3.0100^***^	0.0013

### Panel Co-integration

To examine co-integration test statistics, we applied Kao test (33). The statistical results indicate existence of strong panel co-integration among number of patients reported, income level, education, and population density as shown in Table 2. To further validate our results for robustness, we applied Pedroni test which shows the panels are co-integrated with the exception of Modified Philips-Peron test.

**Table 2:** Results of Kao and PedroniCo-integration test

***Variable***	**t*-statistic***	**P*-value***
Modified Dickey-Fuller t	−5.8461^***^	0.0000
Dickey-Fuller t	−5.6047^***^	0.0000
Augmented Dickey-Fuller t	−2.5498^***^	0.0054
Unadjusted Modified Dickey-Fuller t	−9.3013^***^	0.0000
Unadjusted Dickey-Fuller t	−6.3698^***^	0.0000
Modified Phillips-Perron t	1.0542	0.1459
Phillips-Perron t	−4.1550^***^	0.0000
Augmented Dickey-Fuller t	−3.8366^***^	0.0001

**Note:** The statistical significance at 1%, 5%, 10% is denoted by ***, **, * respectively

### Long and Short Run Estimation Results

The Hausman test result for long run homogeneity, which allows one to choose between the MG and PMG estimators (34, 35), is shown in Table 3. The estimated result shows that PMG estimator is better than MG estimator as p-value is 0.5483, and is greater than 0.05;therefore, we preferred PMG estimator as it gives more efficient results. The long run coefficients under the PMG estimator are more efficient and consistent than MG estimator because MG technique deals with averaging the estimates of individual regressions while PMG technique permits the short run error variances and coefficients to differ among countries (31).

**Table 3:** Results of Hausman test

***Coefficients***				
	(b)	(B)	(b-B)	sqrt(diag(V_b-V_B))
	Mg	Pmg	Difference	S.E.
Patients	−0.2970	−0.2481	−0.0490	0.5940
Pop	−0.1012	0.0094	−0.1106	0.2303
Temp	0.8377	0.3108	0.5269	1.1617
Temp^2^	−2.6983	−1.2045	−1.4938	4.6571
Gdp	−19.1733	1.8186	−20.9919	16.3932
Led	17.7367	−1.9991	19.7358	16.1565
b consistent under Ho and Ha; obtained from MG estimation, B inconsistent under Ha, efficient under Ho; obtained from PMG estimation: Ho: difference in coefficients not systematic				
χ^2^(6) = (b-B)'[(V_b-V_B)^(−1)](b-B) = 4.96				
Prob>χ^2^ = 0.5483				

The PMG estimator shows the coherence and asymptotic properties of mixed of the series with mixed order of integration. PMG exhibits the long-run relationship and gives ECM coefficient which further confirms the co- integration of the variables.

Table 4 shows the estimated long and short run elasticity of patients with respect to temperature, population density, education and income growth. Coefficient of the error correction is negative and significant, which indicates that the model is stable and convergence to long run values, in response to a shock, occurs quickly.

**Table 4:** PMG estimation results

***Dependent variable Patients***	***Coefficient***	***Std. Err.***	***t-statistic***	***Prob***
		Long run estimation		
Patients	−2.4807	0.0955	−2.60^***^	0.009
Pop	0.0094	0.0034	2.79^***^	0.005
Temp	0.3107	0.1659	1.87^*^	0.061
Dlgdp	1.8186	1.9105	0.95	0.341
Led	−1.9991	0.4073	−4.91^***^	0.000
Short run estimation—full sample				
ECT	−0.7552	0.1593	−4.74^***^	0.000
Pop	−0.1128	0.8186	−0.14	0.890
Temp	0.1017	0.2091	0.49	0.627
Dlgdp	3.2642	4.4032	0.74	0.458
Led	0.1712	1.8963	0.09	0.928

**Note:** The statistical significance at 1%, 5%, 10% is denoted by ***, **, * respectively

The long run results indicate positive and significant association between temperature and number of patients. This result is consistent with previous studies (6, 7, 36–39). It can be inferred that increase in temperature would lead to increase in water temperature which would subsequently boost the growth of copepods, zooplankton, phytoplankton or algal blooms and consequently would increase the survivability of *V. cholera* and consequent increase in the cholera incidence (20, 36, 40).

At the same time higher temperature may lead to increasing sea level and the resulting intrusion of seawater to land, especially in offshore areas increasing the survivability of *V. cholera* corresponded by swift growth in absence of public health measures (40). Moreover, the cholera cannot be labeled as a simple calculation of pathogens and human host, but it is based on a complex network like weather patterns, water reservoirs, zooplankton, phages and communal behavior of surface-attached cells (20). Similarly, in case of measles the disease spreads due to a change in human habitation and climate change along with other reasons like fluctuation in temperature indirectly promoting the measles’ spread because climate change stimulates the migration and consequent higher population density in safer cities (25).

Our empirical results prove variables like income and population density having a positive and statistically significant effect on incidence of infectious diseases, while endorse the effect of education as negative. It is generally assumed that income and patient ratio depicts a quadratic association i.e. inverted U-shaped elaborating that as income increases, the possibility of infectious diseases increases too. Albeit, after a certain income level, number of patients decreases with increase in income. The increase in income will increase the possibility of more investment in industrial sector correspondingly worsening the environment as in the case of environmental and health Kuznets curve. The deterioration in environment helps the cholera bacteria to flourish and consequent outbreak of more cases.

While high income ensures affordable access to medication implicitly assisting the healthcare professionals to note the reported cases which are otherwise not possible due to low income (30).These results further re-confirm the negative relationship between education and incidence of infectious diseases (cholera, measles) patients as was suggested in previous studies (30, 41). It explains that education provides more knowledge and awareness to common people about the diseases and their cause and prevention and thus has limited chances of widespread infectious diseases. The results also verify the positive relationship between population density and the number of patients. The higher concentration of population facilitates the chances of outbreak of cholera and measles (30). The outbreak of these diseases remains a significant risk factor in highly densely populated areas mostly in developing countries having limited access to clean water and sanitation services.

The regions with higher population density, proximity to surface water and lower level of education are associated with higher risk of cholera motility and morbidity (41). Thus, education in this context can provide them a better understanding on how to take care of their community and household for preventive measures. Although, public health policies may reduce the impact, however, relying solely on public health measures is not adequate, we need to understand that slums prevalent in developing countries is a major cause of the higher population density and hence higher incidence of infectious diseases. It is therefore, essential to improve as well as implement town planning policies that could reduce higher population density.

### Panel Causality Testing Results

Presence of a long run panel co-integration among infectious diseases, climate change, education, income and population density imply the presence of Granger causality as shown in Table 5.

**Table 5:** Results of Panel Causality test

***Causalities***	***w-statistics***		**P*-value***	
patients⟺temp	2.1985^**^	2.7813^***^	0.02	0.005
patients⟺gdp	0.9013	−0.7730	0.360	0.430
patients⟺pop	9.3106^***^	6.4978^***^	0.000	0.000
patients⟺led	7.0436^***^	4.9759^***^	0.000	0.000

**Note:** The statistical significance at 1%, 5%, 10% is denoted by ***, **, * respectively

Results verify the two-way causality among infectious diseases, climate change, education, and population density while no long run causality has been found in case of income. The results verify the impacts of climate change on the prevalence of diseases in the countries hard hit by the climate changes in recent times. Our results are consistent with other studies (42–44), who concluded the uni-directional causality between climate change and infectious disease.

## Conclusion

Temperature has a positive relationship with the number of patients affected from infectious diseases. Similarly, population density had similar effects on patients; however, income was statistically insignificant. We found negative impact of education with reference to the number of patients where the increase in awareness will facilitate the people to take preventive measures to avoid infectious diseases.

The investment in the public health sector will result in to decrease the number of patients reported of infectious diseases in the long run. Therefore, proper adaptation and mitigation policies are designed to overcome the impacts of climate change on health. The policies may include improving awareness through education, use of technology in weather forecasting and warning system, disaster management vigilance system, proper medication and better town planning policies.

## Ethical considerations

Ethical issues (Including plagiarism, informed consent, misconduct, data fabrication and/or falsification, double publication and/or submission, redundancy, etc.) have been completely observed by the authors.
